# Tracheal Tube-Mounted Camera Assisted Intubation vs. Videolaryngoscopy in Expected Difficult Airway: A Prospective, Randomized Trial (VivaOP Trial)

**DOI:** 10.3389/fmed.2021.767182

**Published:** 2021-12-15

**Authors:** Jörn Grensemann, Emma Möhlenkamp, Philipp Breitfeld, Pischtaz A. Tariparast, Tanja Peters, Mark A. Punke, Stefan Kluge, Martin Petzoldt

**Affiliations:** Center of Anesthesiology and Intensive Care Medicine, University Medical Center Hamburg-Eppendorf, Hamburg, Germany

**Keywords:** airway management (MeSH), intubation (intratracheal), respiration (artificial), laryngoscopy, laryngoscope and intubation, VivaSight

## Abstract

**Background:** Tracheal intubation in patients with an expected difficult airway may be facilitated by videolaryngoscopy (VL). The VL viewing axis angle is specified by the blade shape and visualization of the larynx may fail if the angle does not meet anatomy of the patient. A tube with an integrated camera at its tip (VST, VivaSight-SL) may be advantageous due to its adjustable viewing axis by means of angulating an included stylet.

**Methods:** With ethics approval, we studied the VST vs. VL in a prospective non-inferiority trial using end-tidal oxygen fractions (etO_2_) after intubation, first-attempt success rates (FAS), visualization assessed by the percentage of glottis opening (POGO) scale, and time to intubation (TTI) as outcome parameters.

**Results:** In this study, 48 patients with a predicted difficult airway were randomized 1:1 to intubation with VST or VL. Concerning oxygenation, the VST was non-inferior to VL with etO_2_ of 0.79 ± 0.08 (95% CIs: 0.75–0.82) vs. 0.81 ± 0.06 (0.79–0.84) for the VL group, mean difference 0.02 (−0.07 to 0.02), *p* = 0.234. FAS was 79% for VST and 88% for VL (*p* = 0.449). POGO was 89 ± 21% in the VST-group and 60 ± 36% in the VL group, *p* = 0.002. TTI was 100 ± 57 s in the VST group and 68 ± 65 s in the VL group (*p* = 0.079). TTI with one attempt was 84 ± 31 s vs. 49 ± 14 s, *p* < 0.001.

**Conclusion:** In patients with difficult airways, tracheal intubation with the VST is feasible without negative impact on oxygenation, improves visualization but prolongs intubation. The VST deserves further study to identify patients that might benefit from intubation with VST.

## Introduction

Tracheal intubation is required for mechanical ventilation during general anesthesia and to prevent aspiration of secretions. Commonly, direct laryngoscopy (DL) is used, but this technique may fail in patients with a difficult airway (1), e.g., in patients scheduled for otorhinolaryngologic or oral and maxillofacial surgery. Besides intubation with a bronchoscope that is still regarded as the gold standard, videolaryngoscopy (VL) has evolved as a valuable alternative technique in patients with an expected or unexpected difficult airway (2–4). However, VL has some limitations and may fail due to insufficient visualization of the larynx or impossible tube advancement. Recently, a tracheal tube with an integrated camera has been introduced (VST, VivaSight-SL, Ambu A/S, Ballerup, Denmark) that may allow for direct guidance of the tube and may aid in tracheal intubation in patients with difficult airways (5). This tube has been evaluated under the clinical conditions in intensive care patients (6) and patients with morbid adiposity compared with DL (7), but there is a paucity of data in patients with difficult airways, so far. As opposed to VL, the camera axis of VST may be adjusted individually by modifying the angulation of a stylet and by direct steering of the tip of the tube during tube advancement which may provide a benefit in the cases of difficult airways.

Therefore, we assessed the feasibility of intubation with the VST in patients with a predicted difficult airway compared with VL in a prospective randomized non-inferiority trial. Due to the paucity of data in these patients, we chose end-tidal oxygen fractions (etO_2_) after intubation as an important safety parameter for the primary outcome measure and to ensure adequate oxygenation throughout the intubation procedure.

## Methods

### Ethics

The study was approved by the Ethics Committee of the Hamburg Chamber of Physicians (PV7276, June 6, 2020, chairman Prof. Dr. Carstensen). All patients provided written informed consent. The study was conducted in accordance with the Declaration of Helsinki, adheres to the current CONSORT guideline, and was registered prior to patient enrolment with ClinicalTrials.gov (NCT04501692) on August 6, 2020.

### Study Design

The VivaOP trial was a prospective randomized non-inferiority trial with a 1:1 allocation ratio conducted in the Center of Anesthesiology and Intensive Care Medicine at the University Medical Center, Hamburg-Eppendorf, Germany.

### Eligibility

Patients were eligible if they were at least 18 years old, required transoral tracheal intubation for elective ear, nose, and throat (ENT), or oral and maxillofacial (OMF) surgery, and had an expected difficult airway. To assess airway difficulty, all patients received a structured preoperative airway risk evaluation in line with the standards of the Department of Anesthesiology, such as physical examination, medical history, assessment of the Simplified Airway Risk Index (SARI) (8, 9), Wilson score (10), upper-lip-bite-test (11), and transnasal videoendoscopy, if appropriate. Individuals with an expected difficult airway as rated by the responsible anesthetist in the Pre-assessment Clinic were included while patients with a verified indication for awake tracheal intubation (e.g., via a bronchoscope), transnasal tracheal intubation, rapid-sequence induction, and loose teeth were excluded.

### Participating Physicians

Physicians with a specialization in airway management, i.e., anesthesiology specialists and experienced fellows were chosen as participators and their duration of work experience recorded. All participating anesthetists were trained with the VST in a structured manikin airway training to avoid bias due to insufficient skill level with this device.

### Interventions

**Figure 1:**
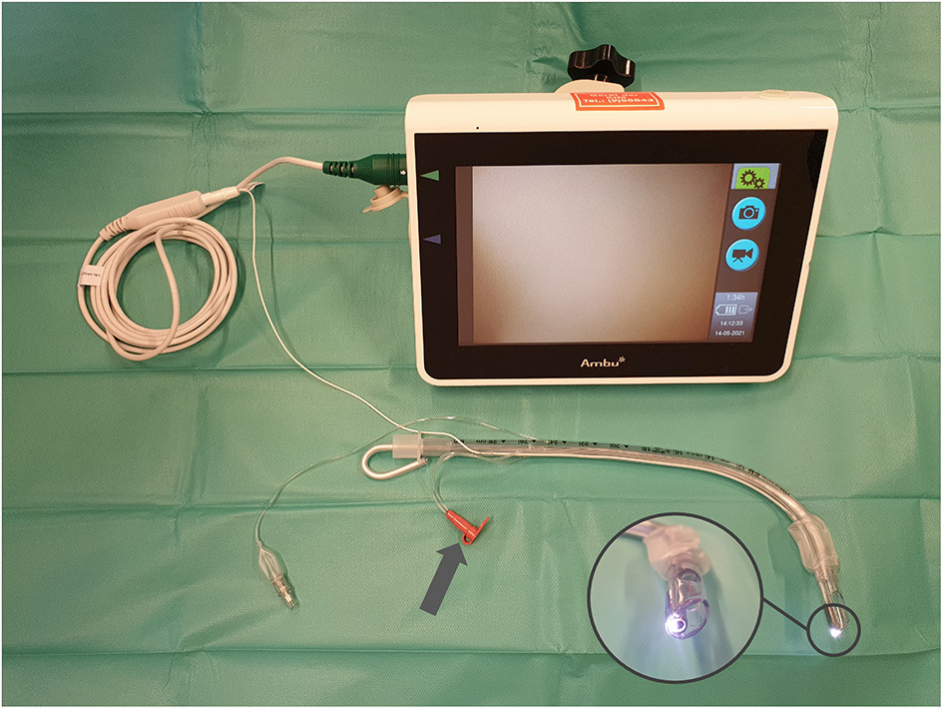
Depiction of the setup of VivaSight-SL tubes. Prepared “optimal” stylet angulation achieved by superposition of 60 intubations in a pretrial manikin training (prepared for difficult airways) by 15 operators. VivaSight-SL tube connected to aView monitor; arrow indicates camera rinsing port; inset depicts camera of tube.

Patients randomized to the intervention group received tracheal intubation with a VST. Depending on gender, size of the patient, and planned surgery, tubes with inner diameters of 7.0, 7.5, and 8.0 were available. The tubes camera was connected to an Ambu aView monitor (Ambu A/S, Ballerup, Denmark). The VST with its included rigid stylet was pre-formed to a predetermined standardized angle obtained by prior trials on a manikin set up for a difficult airway and could be modified by the intubating anesthetist. A depiction of the setup and the curvature is provided in Figure 1. To prevent from soiling of the camera by secretions or mucosal contact, the tongue was elevated with a Macintosh type laryngoscope.

Patients randomized to the control group received tracheal intubation with a C-MAC videolaryngoscope (Karl Storz SE & Co. KG, Tuttlingen, Germany) with a size 3 or 4 Macintosh type blade.

Anesthesia management, the choice of the blade and tube size, as well as the use of adjuncts, such as stylets, introducers, or forceps or airway optimization maneuvers as backward upward rightward pressure (BURP) or optimum external laryngeal manipulation (OELM) were at the discretion of the responsible anesthetist. The tube size was chosen prior to randomization. All intubations were recorded through the respective monitors for later review.

Pre-oxygenation was performed for 5 min with a tight sealing face mask connected to the anesthesia rebreathing circuit (Perseus A500 Anesthesia Workstation, Drägerwerk, Lübeck, Germany). The circuit was flushed before the beginning of pre-oxygenation and an oxygen flow of 15 L/min was maintained throughout.

### Outcome Parameters

The primary outcome measure was the lowest end-tidal fraction of oxygen within 2 min after intubation (12). Secondary outcome parameters were the first attempt success rate, the overall success rate with a failure defined as transition to a different device or VL blade type (hyperangulated blade), time to successful intubation, time to successful intubation with one attempt, and total and average number of attempts. Furthermore, the end-tidal carbon dioxide (etCO_2_) after intubation and Cormack-Lehane grade (13) were obtained. The percentage of glottis opening (POGO) scale (14) was measured from the recorded videos from the frame with the largest glottis orifice area. Intubation difficulty, quality of glottis visualization, and ease of tube advancement were rated on visual analog scales (0–100, lower values better). The complications during intubation as regurgitation, aspiration, hypotension (systolic blood pressure below 70 mmHg), and hypoxia (pulse oximetric saturation below 80%) were recorded (Infinity Delta vital signs monitor, Drägerwerk AG, Lübeck, Germany). Time to successful intubation was measured from the laryngoscope blade passing the teeth to the first of at least three positive, non-declining etCO_2_ readings (no visual decrease in capnography) obtained by side stream capnography (Perseus A500 Anesthesia Workstation, Drägerwerk AG, Lübeck, Germany).

### Sample Size

*A priori* power analysis indicated a required sample size of 48 patients randomized 1:1 to either VST or VL. This calculation was based on an expected end-tidal fraction of oxygen after intubation of 80% with a SD of 8%, and a non-inferiority margin of 10%, with errors of α = 0.025 and β = 0.2 (PASS version 08.0.6, NCSS, LLC. Kaysville, UT, USA) (12).

### Randomization

Patients were randomized in the operating theater immediately prior to anesthesia induction and after the anesthetist was assigned to the patient and the anesthetist had chosen the required tracheal tube diameter as well as the desired laryngoscope blade size. The randomization codes were obtained from sealed opaque envelopes.

### Statistics

Microsoft Excel 2019 (Microsoft Corp., Redmond, WA, USA) was used for data management and the SPSS statistical software package (version 25, IBM Inc., Armonk, NY, USA) was used for statistical analysis. The *t*-tests and contingency tables with chi-square and Fisher's tests were used, as applicable. Two-tailed *p* < 0.05 were regarded as statistically significant.

## Results

**Figure 2:**
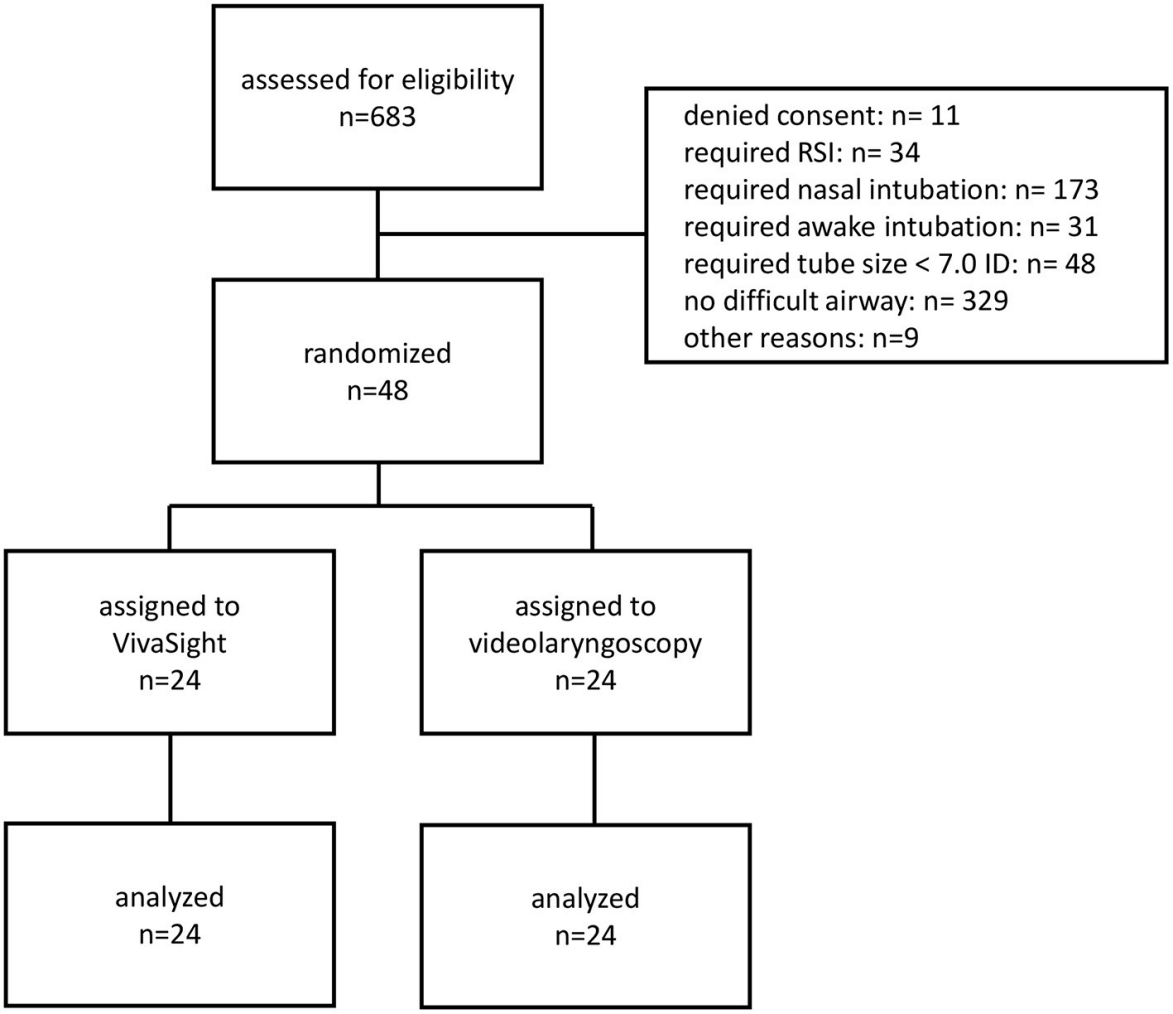
CONSORT diagram. RSI, rapid sequence induction.

From August 27, 2020, to February 12, 2021, 48 patients receiving tracheal intubation were randomized to either camera-assisted intubation with the VST or intubation with VL in a 1:1 ratio (as shown in Figure 2). The baseline characteristics of patients are shown in Table 1. Groups had similar values for baseline saturation of oxygen, and end-tidal fractions of oxygen and carbon dioxide after pre-oxygenation (as shown in Table 2). All patients received intubation in deep anesthesia and were paralyzed.

**Table 1:** The patient characteristics and airway conditions.

**Parameter**		**VivaSight-SL** ***n* = 24**	**Videolaryngoscopy** ***n* = 24**
Age [years]		63 ± 15	57 ± 16
Sex	Male	18	16
	Female	6	8
Weight [kg]		88 ± 23	77 ± 21
Height [cm]		175 ± 10	174 ± 10
ASA	1	0	1
	2	9	12
	3	15	11
History of difficult airway	None	9	9
	Possible	5	4
	Yes	10	11
SARI score [0–12]		5.4 ± 2.0	5.0 ± 1.9
Wilson score [0–10]		4.3 ± 1.3	4.1 ± 1.2
Thyromental distance [cm]		7.9 ± 1.3	7.6 ± 1.5
Mouth opening [cm]		4.1 ± 1.0	4.0 ± 1.7
Mandibular protrusion	Impossible	5	5
	Restricted	9	3
	Unrestricted	10	16
Neck mobility	Above 90°	2	4
	90° to 80°	9	10
	Below 80°	13	10
Mallampati score	1	0	1
	2	1	4
	3	16	11
	4	7	8
Upper lip bite test	Class 1	6	8
	Class 2	13	9
	Class 3	5	6
Retrognathia	None	13	13
	Moderate	8	4
	Severe	3	7
Dysmorphia	Face	14	10
	Mouth	15	17
	Throat	11	10
Tumor	Larynx	6	4
	Pharynx	5	4
History of radiotherapy		9	8
Protruding teeth		0	6
Maxillary joint anomalies		9	10
Stridor		1	2
Dyspnea		3	3
Dysphonia		15	14
Dysphagia		14	10

*Data are shown as mean ± SD or numbers, as applicable. SARI: simplified airway risk index. Upper lip bite test score: class 1 indicates lower incisors can bite upper lip above vermilion line, class 2 indicates lower incisors can bite upper lip below vermilion line, and class 3 indicates lower incisors cannot bite upper lit*.

**Table 2:** Outcome parameters.

**Parameter**	**VivaSight-SL** ***n* = 24**	**Videolaryngoscopy** ***n* = 24**	***p***
First attempt success rate	18/24	21/24	0.267
Overall success rate	24/24	23/24	0.312
Total number of attempts	1 attempt: 21	1 attempt: 21	0.392
	2 attempts: 3	2 attempts: 1	
		3 attempts: 1	
		>3 attempts: 1 ^*^	
Time to successful intubation [s]	100 ± 57	68 ± 65	0.079
Time to successful intubation with one attempt [s]	84 ± 31	49 ± 14	<0.001
End-Tidal oxygen fraction after pre-oxygenation	0.82 ± 0.06	0.82 ± 0.04	0.978
End-Tidal carbon dioxide after pre-oxygenation [mmHg]	31 ± 7	32 ± 5	0.689
Lowest end-tidal oxygen fraction within 2 min after intubation	0.79 ± 0.08	0.81 ± 0.06	0.234
Highest end-tidal carbon dioxide within 2 min after intubation [mmHg]	39 ± 7	37 ± 7	0.277
POGO [%]	89 ± 21	60 ± 36	0.002
Cormack-Lehane	1: 15	1: 2	<0.001
	2a: 5	2a: 17	
	2b: 4	2b: 2	
	3: 0	3: 2	
	4: 0	4: 1	
Overall difficulty of airway management rated on VAS [0–100]	32 ± 24	26 ± 29	0.431
Visualization rated on VAS [0–100]	25 ± 26	19 ± 27	0.481
Tube advancement difficulty rated on VAS [0–100]	47 ± 23	21 ± 25	0.001
Regurgitation/aspiration during intubation	None	None	n/a
Accidental esophageal intubation	None	None	n/a
SpO_2_ <80%	1/24	0/24	0.312
SpO_2_ [%]	99 ± 2	99 ± 2	0.605
Systolic blood pressure <70 mmHg	4/24	1/24	0.156

*Data are shown as mean ± SD, numbers, or proportions, as applicable. POGO: percentage of glottis opening scale; VAS: visual analog scale for rating of difficulty from 0 to 100, lower values better*.

* *Unsuccessful after three attempts and method changed to hyperangulated blade*.

**Figure 3:**
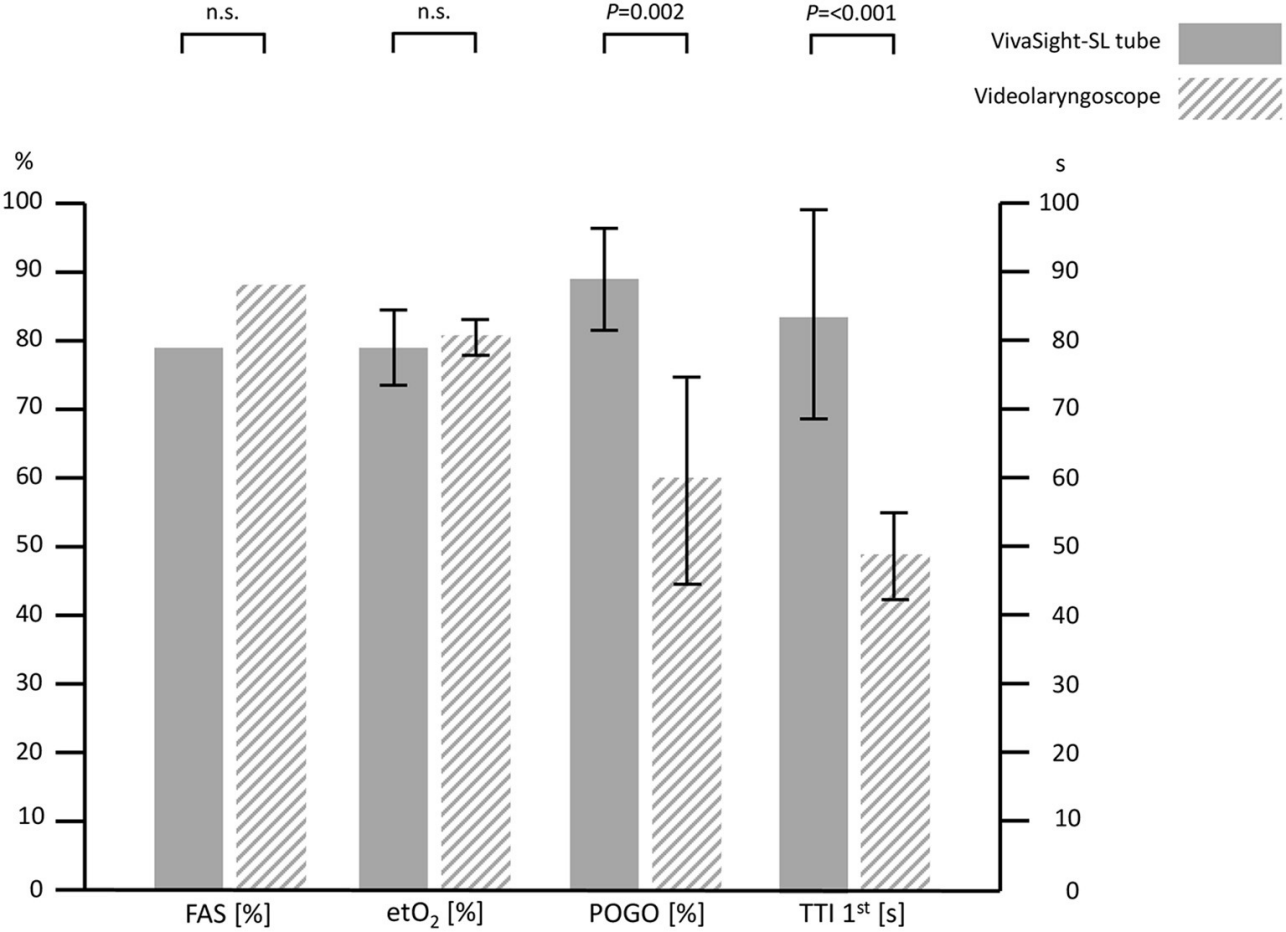
Overview of success rate, oxygenation, visualization, and duration for VivaSight and videolaryngoscopy. FAS, first attempt success rate; etO_2_, end-tidal oxygen fraction after intubation; POGO, percentage of glottis opening scale; TTI 1st, time to intubation with success in the first attempt. Error bars indicate 95% CIs; n.s., not statistically significant.

Concerning the oxygenation after airway management, intubation with the VST was non-inferior to VL with a post-intubation fraction of oxygen of 0.79 ± 0.08 (95% CIs: 0.75–0.82) in the VST group versus 0.81 ± 0.06 (0.79–0.84) in the VL group with a mean difference of 0.02 (95% CIs: −0.07; 0.02), *p* = 0.234. The first attempt success rate was 79% in the VST group and 88% in the control group, *p* = 0.449. The overall success rate in the VST group was 100 and 96% in the VL group (*p* = 0.312) with one patient requiring a laryngoscopy with a hyperangulated blade. POGO was 89 ± 21% in the VST-group versus 60 ± 36% in the VL group, *p* = 0.002. While time to successful intubation did not statistically differ between the groups, time to successful intubation in the first attempt was prolonged in the VST group (84 ± 31 s vs. 49 ± 14 s, *p* < 0.001). A graphical depiction is given in Figure 3. Tube advancement was rated to be more difficult for the VST (visual analog scale 0–100, lower values better: 47 ± 23 vs. 21 ± 25, *p* = 0.001). Other parameters did not differ between the groups. Regurgitation, aspiration, or accidental esophageal intubation did not occur in any patient. An overview on results is given in Table 2. Anesthetists professional experience was 16 ± 5 years in the VST and 15 ± 5 years in the VL group, *p* = 0.277. The pre-trial VST angulation studies with a manikin yielded an angle of 85 degrees. Results of an evaluation of this setup in a manikin trial are reported in Supplementary Table 1.

## Discussion

In this prospective randomized trial comparing tracheal intubation assisted by a tube-mounted camera (VST) with intubation by VL in patients with a predicted difficult airway, we found no difference concerning oxygenation during airway management or the first attempt success rate. While time to intubation with the VST was prolonged compared with VL, visualization of the larynx by the VST was improved as compared with VL. However, tube advancement was rated more difficult using the VST. No significant differences were found for other parameters, particularly not for complications, such as hypoxia, hypotension, regurgitation, or accidental esophageal intubation.

So far, the VST has been evaluated for tracheal intubation in patients with a predicted non-difficult airway in intensive care (6), in patients with morbid obesity (7), via supraglottic airway devices (15, 16), in manikins (17–21), in a cadaver study (22), and for the guidance of percutaneous dilatational tracheostomy (23, 24).

Awake intubation *via* bronchoscopy is often regarded as the gold standard technique for the management of the expected difficult airway (3), but this method may be complex, difficult to learn, expensive, time-consuming, and requires thorough patient preparation. Awake videolaryngoscopy has been found to be a safe and effective alternative in carefully selected patients (25). Videolaryngoscopy plays a key role in unexpected difficult airways (4, 26), but the angle of the attached camera is fixed in both Macintosh style and hyperangulated blades. Therefore, visualization of the larynx may result in inadequate viewing conditions. In particular, a restricted epiglottis motion with the epiglottis being adherent to the pharynx [grade 3 view according to Cook (27)] is a pitfall and possible limitation of VL and rather a domain of flexible intubation methods (28). Beyond bronchoscopic and videolaryngoscopic intubation techniques further video-guided techniques using tube or stylet mounted cameras have been introduced but their role for the management of the expected or unexpected difficult intubation remains unclear (29).

In cases of insufficient visualization of the larynx for intubation, a hyperangulated blade or flexible bronchoscopy may be used to obtain visualization of the larynx with a more advantageous viewing angle. With the VST, the axis of the camera may be individually adjusted by angulation of the stylet, presumably explaining the improved laryngeal viewing conditions with a higher POGO score for the VST found in our study. For visualization during VL, it has been shown that insufficient laryngeal views above Cormack/Lehane grade 2 are associated with an increased failure rate of the method (30). Failure rates for VL depend on the type of VL, as well, with first pass success rates ranging from below 40% to above 90% (31). Although visualization was superior for the VST group in our study, we could not show a decrease in failure rate. Interestingly, the most common cause for failure of intubation with VL is the inability to direct the tube toward the laryngeal inlet (32). Presumably, the VST provides a benefit in this respect as the camera provides direct guidance.

The number of intubation attempts did not differ in our study. Previously, more than two attempts have been associated with an increase in complications (33) and strategies have been suggested to increase the rate of first attempt success of intubation, one strategy focusing on the selection of an adequate device for the respective predicted difficult airway (34).

For VL, an overall intubation time of approximately 50–60 s has been reported in human subjects with a difficult airway (31). For the VST, approximately 30 s have been reported previously, but these results were obtained in patients without difficult airways (6). This contrasts with the required duration for intubation in our study with the VST needing nearly three times longer, only counting intubations that were successful on first attempt. We could not find any negative effects as desaturation or a higher rate of hypotension in our patients, but we presume that the prolonged intubation increases complications in patients prone to hypoxia, i.e., in critically ill patients or patients with morbid obesity (35, 36). This may limit the usefulness of the VST to elective cases without risk for hypoxia.

We chose to use a Macintosh-style VL for the control group, angulated at approximately 30 degrees, while hyperangulated blades are angulated at approximately 60 degrees (28, 37). For the VST, we used an angulation of approximately 85 degrees as obtained prior to the beginning of our study that was more pronounced than typical hyperangulated laryngoscope blades. For VL with hyperangulated blades, laryngoscopic view is often improved compared with Macintosh blades, but a common problem arises when attempting to advance the tube through the laryngeal inlet because the tube may hit the anterior tracheal wall at a nearly perpendicular angle, hindering further tube advancement (37), explaining why tube advancement in our study was rated more difficult with the VST as compared with VL.

Recently, a video stylet inserted into a tracheal tube providing camera guidance has been evaluated for intubation in patients with cervical immobilization showing a significantly lower first attempt success rate and prolonged time to intubation as compared with VL (29). As the basic principles of intubation with camera guidance resemble the VST approach, and the results are comparable with our study results, tube-camera guided intubation might indeed be inferior to a laryngoscope approach in the clinical settings, but further data are required.

Our study has the following limitations. Our choice of calculating the sample size for etO_2_ as the primary endpoint may be questionable as first attempt success rates are widely accepted as endpoints for studies evaluating airway devices. However, sufficient oxygenation is paramount in airway management and an important safety parameter and thus chosen purposely. Our sample size was adequately powered to show non-inferiority for oxygenation but may be underpowered for the first attempt success rate. However, we provide the first data in human subjects for use of the VST in the predicted difficult airways. The learning curve for the VST may not have had reached its peak after the manikin training, but all participating anesthetists were highly experienced in airway management, such as the management of predicted and unexpected difficult airway scenarios.

## Conclusion

Tracheal intubation with the VST in patients with an expected difficult airway is feasible, and oxygenation was not inferior as an important safety parameter in the VST group. The first pass success rate in our cohort did not differ between groups. Visualization of the larynx with the VST was superior to VL but time to intubation was prolonged with the VST. We believe further studies are warranted to define which cohorts of patients might benefit from intubation with the VST.

## Data Availability Statement

The raw data supporting the conclusions of this article will be made available by the authors, without undue reservation.

## Ethics Statement

The studies involving human participants were reviewed and approved by Ethics Committee of the Hamburg Chamber of Physicians (No. PV7276, June 6, 2020). The patients/participants provided their written informed consent to participate in this study.

## Author Contributions

JG designed the study, wrote the manuscript, performed the statistical analysis, and interpreted the data. EM recruited the patients, acquired the data, helped to write the manuscript, and helped with the statistical analysis. PB and TP helped to recruit the patients and to acquire the data. PT helped with the statistical analysis and to interpret the data. MAP helped to acquire the data and to revise the manuscript. SK helped to interpret the data and to revise the manuscript. MP designed the study, helped to acquire the data, interpreted the data, and revised the manuscript. All authors read and approved the final manuscript.

## Funding

This study was funded exclusively from the departmental resources including the acquisition of the VivaSight-SL tracheal tubes.

## Conflict of Interest

JG has received research support from Adroit Surgical, Ambu, ETView, and Infectopharm, and received consultant and lecture fees from Drägerwerk, Fresenius Medical, GE Healthcare, and Smith Medical; SK received research support from Ambu, Daiichi Sankyo, ETView Ltd., Fisher & Paykel, Pfizer, and Xenios, lecture fees from Astra, C.R.Bard, Baxter, Biotest, Cytosorbents, Daiichi Sankyo, Fresenius, Gilead, Mitsubishi Tanabe Pharma, MSD, Pfizer, Philips, and Zoll, and consultant fees from Bayer, Fresenius, Gilead, MSD, and Pfizer; MP received a research grant awarded by Verathon. The remaining authors declare that the research was conducted in the absence of any commercial or financial relationships that could be construed as a potential conflict of interest.

## Publisher's Note

All claims expressed in this article are solely those of the authors and do not necessarily represent those of their affiliated organizations, or those of the publisher, the editors and the reviewers. Any product that may be evaluated in this article, or claim that may be made by its manufacturer, is not guaranteed or endorsed by the publisher.
