# Supplementary material for: Tracheal Tube-Mounted Camera Assisted Intubation vs. Videolaryngoscopy in Expected Difficult Airway: A Prospective, Randomized Trial (VivaOP Trial)
Source: Front Med (Lausanne). 2021 Dec 15;8:767182. doi: 10.3389/fmed.2021.767182 (PMC8714897; doi:10.3389/fmed.2021.767182)
Supplement: Supplementary file 1 [file Data_Sheet_1.PDF]

The setup of VivaSight-SL tubes with the integrated stylets angulated at 85° was tested in a manikin trial. Three manikins, AirSim Multi, AirSim Advance X and AirSim Broncho CombiX (TruCorp Ltd., United Kingdom) were prepared with bandages, restricted reclination and enlarged tongue to simulate a difficult airway. Each manikin was intubated with the VivaSight-SL (VST, Ambu A/S, Ballerup, Denmark) and by videolaryngoscopy (C-MAC, Macintosh type blade 3, Karl Storz SE & Co. KG, Tuttlingen, Germany). We recorded first attempt success rates (FAS), overall success rate, number of attempts, time to intubation (TTI), the percentage of glottis opening score (POGO), and Cormack-Lehane grades (CL). Furthermore, difficulty of visualization, intubation and overall difficulty were assessed with visual analogue scales (VAS, 0-100, lower values easier). Two-sided t-tests and contingency tables were used for statistical comparison (SPSS statistical software package, version 25, IBM Inc., Armonk, NY, USA).

Twelve consultant anesthetists with a mean occupational experience of 13 ± 5 years participated in this study. An overview on results is given in table S1.

Table S1: Overview on results of manikin evaluation:

	VivaSight-SL	Videolaryngoscopy	P
FAS (%)	78%	94%	.04
overall success rate (%)	97%	100%	.32
Mean number of attempts (n)	1.4 ± 0.9	1.1 ± 0.2	.03
TTI (s)	48 ± 43	31 ± 29	.06
POGO (%)	66 ± 32	53 ± 31	.09
CL (n)	°1: 12<br>°2: 16<br>°3: 4<br>°4: 3<br>n/a: 1	°1: 3<br>°2: 25<br>°3: 5<br>°4: 3	.15
Difficulty of visualization	43 ± 33	42 ± 30	.87
Difficulty of intubation	47 ± 28	39 ± 29	.29
Overall difficulty	44 ± 28	40 ± 29	.50

Data are given as mean ± standard deviation. FAS: first attempt success rate; TTI: time to intubation; POGO: percentage of glottis opening scale; CL: grade according to Cormack and Lehane; n/a: not applicable (due to failure of method).

Although no statistically significant difference could be demonstrated in this associated manikin study, a trend for an improved FAS and fewer mean number of attempts in favor of videolaryngoscopy could be shown. On the other hand, POGO scores were higher in the VST group indicating a better visualization, but TTI was prolonged. In the subjective ratings on VAS, no difference could be shown. Considering the possibly improved visualization with the VST, these results support the conduct of a similar study in human subjects.
